# Elucidating photoreceptor gene function with in-vivo CRISPR knock-out perturbation assay

**DOI:** 10.21203/rs.3.rs-7670521/v1

**Published:** 2025-10-27

**Authors:** Riccardo Sangermano, Egle Galdikaite-Braziene, Kinga M. Bujakowska

**Affiliations:** Massachusetts Eye and Ear, Harvard Medical School; Massachusetts Eye and Ear, Harvard Medical School; Massachusetts Eye and Ear, Harvard Medical School

## Abstract

Over the past two decades, considerable progress has been made in cataloguing genes and active chromatin elements in humans. However, despite these efforts, less than a quarter of genes have been assigned a function in the context of human disease, which limits our ability to interpret clinical genome sequencing results. In the field of inherited retinal diseases, 30–40% of cases remain genetically undiagnosed. This may be partially due to our limited understanding of the function of most retina-expressed genes, which prevents the correct interpretation of their sequence variations. In the effort of elucidating retinal gene function, we aimed at developing a protocol for *in-vivo* CRISPR-based gene knock-out perturbation in the mouse retina. This methodology can be useful to study retinal biology in health and disease, to investigate the effects of ablation of novel uncharacterized genes, and to study possible genetic modifiers of retinal phenotypes.

## INTRODUCTION

Since the release of the first draft of the human genome^1^, substantial progress in genomic research has been made with the classification of ~ 20,000 protein-coding genes, up to 75,000 non-coding genes, and cataloguing common and rare human genome variation^2–8^. There are currently ~ 5,000 genes implicated in Mendelian diseases, and over 3.5 million variants with clinical phenotype interpretations^3,9^. Corresponding phenotypes in other species have been observed in 4,844 genes implicated in Mendelian traits^10^. However, despite these concerted efforts, less than half of the Mendelian disease patients receive a molecular diagnosis after exome or genome sequencing^11,12^. One of the reasons for this low diagnostic rate is that the function of over 75% of genes and their regulatory elements is still poorly understood, which limits our ability to interpret the majority of human genetic variation in disease^11^.

Similar trends are observed in the field of inherited retinal degenerations (IRDs), a group of Mendelian disorders causing blindness in more than 2 million people worldwide^13^. IRDs are highly heterogeneous and can be caused by mutations in one of ~ 300 genes^14^. Yet, 30–40% of IRD cases remain genetically undiagnosed after targeted next-generation sequencing (NGS) approaches or exome sequencing^15–18^. The introduction of genome sequencing in routine diagnostics has brought a limited improvement due to our restricted understanding of the function of the majority of the genes expressed in the retina and of the regulatory regions in the genome^19–21^.

To address this knowledge gap, increasing efforts have focused on characterizing the retinal genome architecture, identifying active chromatin regions, and elucidating gene expression regulation^22,23^. This research is valuable for the understanding of retinal biology and has provided additional diagnoses for IRDs^24–26^. However, an additional and complementary area of research that needs development is the study of the function of genes expressed in the retina, to understand whether their sequence variation may explain some of the missing heritability in IRDs.

Traditionally, an in-depth study of a gene function would be undertaken after a likely causal genetic variant was discovered in human patients or animals with a specific phenotype^27–32^ or through forward genetic screens, such as *N*-ethyl-*N*-nitrosourea mutagenesis in mice^10,33^. However, this approach relies on creating random genetic variation inherited by mouse lines, and thus has a limited throughput and requires large laboratories or consortia to effectively perform a screen of all expressed genes^33^. A faster alternative to that is offered by the CRISPR-based screens, which can be applied in a massively parallel way, as the phenotypic read-out is cell-based rather than whole-organism-based^34–36^.

To investigate retinal gene function in the absence of *in-vitro* models that fully recapitulate the physiological environment of retinal cells^37^, we developed a CRISPR-based knock-out (CRISPR-KO) screening protocol in the mouse retina using lentiviral (LV) vectors and guide (g)RNA libraries. Using IRD genes and genes coding for members of the protein processing and stress response pathways as an example, we demonstrate that this approach can be used to decipher genes that are essential for the photoreceptors. Also, including known genetic modifiers of *Rhodopsin*-associated IRD, we show that our technique can be used to find pathways that can be modulated to alleviate retinal disease. Thus, our approach has great potential for deciphering the biology of the retina in health and disease.

## RESULTS

### Assessment of the lentiviral photoreceptor transduction efficiency and toxicity in-vivo

**Figure 1:**
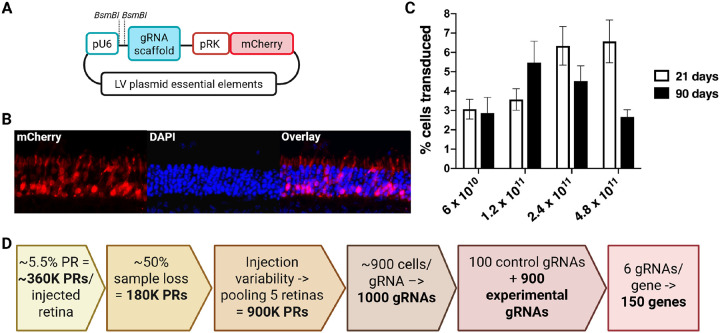
Assessment of the lentiviral photoreceptor transduction efficiency and toxicity *in-vivo*. **A**. Schematic representation of the employed lentiviral (LV) vector expressing the fluorescent mCherry reporter gene under the control of the Rhodopsin Kinase (pRK) promoter. For this experiment, no guide RNAs (gRNAs) were cloned downstream of the U6 promoter; **B**. Cryosection of LV transduced mouse retina showing mCherry positive photoreceptors; **C**. LV transduction efficiency evaluation performed by testing four different LV titers (6×10^10^ vp/ml, 1.2×10^11^ vp/ml, 2.4×10^11^ vp/ml, 4.8×10^11^vp/ml) on an average of 12 pups per titer group and counting the fluorescent transduced cells by fluorescence flow cytometry at an early (PN21) and late (PN90) time point; **D**. Estimated number of cells targeted by one injection and consequent number of genes to include in each lentiviral perturbation library, considering a photoreceptor transduction LV efficiency average of 5.5% (+/−1%).

To establish the feasibility of *in-vivo* CRISPR screening in mouse photoreceptor cells, we first investigated the key limitation of this approach, which is the accessibility of the target cells. Therefore, our first experiment aimed at establishing the optimal conditions for subretinal injections. For this purpose, we used wild-type CD1 mice and LV vectors expressing the fluorescent mCherry reporter gene under the control of the Rhodopsin Kinase (RK) promoter (Fig. 1A–B). Since the LVs transduce replicating cells, we chose post-natal day 3 (PN3) for subretinal injections, as at this early developmental stage, some photoreceptor cells still undergo replication before terminally differentiating^38,39^.

We tested four LVs titers (6×10^10^ vp/ml, 1.2×10^11^ vp/ml, 2.4×10^11^ vp/ml, 4.8×10^11^vp/ml) by injecting an average of 12 pups per titer group and counting the fluorescent transduced cells by fluorescence flow cytometry at an early (PN21) and late (PN90) time point (Fig. 1C). We chose to inject more than 10 mice per group to have a reliable number of mice at the end of each time point, thus compensating for unexpected animal death or retinal tissue losses. For this experiment, no inclusion/exclusion criteria were used. We observed that at PN21, the average transduction efficiency showed a titer-dependent effect, with a transduction efficiency of ~ 6.5% (+/−1%) at the highest titer of 4.8×10^11^vp/ml. However, at the same titer, the average transduction efficiency reduced to ~ 2.7% (+/−0.3%) at PN90, likely due to LV toxicity. The toxic effect was not observed for the two lower titers, with the optimal titer of 1.2×10^11^ vp/ml showing an average transduction efficiency of 3.5 (+/−0.5%) and 5.5% (+/−1%) at PN21 and PN90, respectively. Therefore, we concluded that for *in-vivo* long-term perturbation studies, a LV titer of 1–1.5×10^11^ vp/ml is optimal for maximizing photoreceptor transduction efficiency while avoiding long-term toxicity.

### LV-based in-vivo perturbation influences gRNA library scalability

The average 5.5% (+/−1%) photoreceptor targeting efficiency by LVs enables the calculation of the number of cells targeted by a single injection. Since one mouse retina contains ~ 6,4 million rods and ~ 200,000 cones^40^, ~ 360,000 of them will be transduced in our experimental setup. Due to the likely tissue loss during retinal dissection and gDNA purification, we estimate the recovery of ~ 50% of the transduced material, thus 180,000 cells per retina. Because subretinal injection efficiency can vary between mice, we recommend pooling five injected retinas per sample to reliably obtain ~ 900,000 transduced cells per sample. Targeting 900 cells per gRNA enables consistent detection of its effect, allowing for the inclusion of up to 1000 gRNAs in a single screen^34,41^. Including 100 non-targeting control gRNAs and six gRNAs per gene, permits reliable perturbation of up to 150 genes in one library when retinas from five mice are pooled (Fig. 1D)^34,41,42^.

### CRISPR knock-out screen experimental design

**Figure 2:**
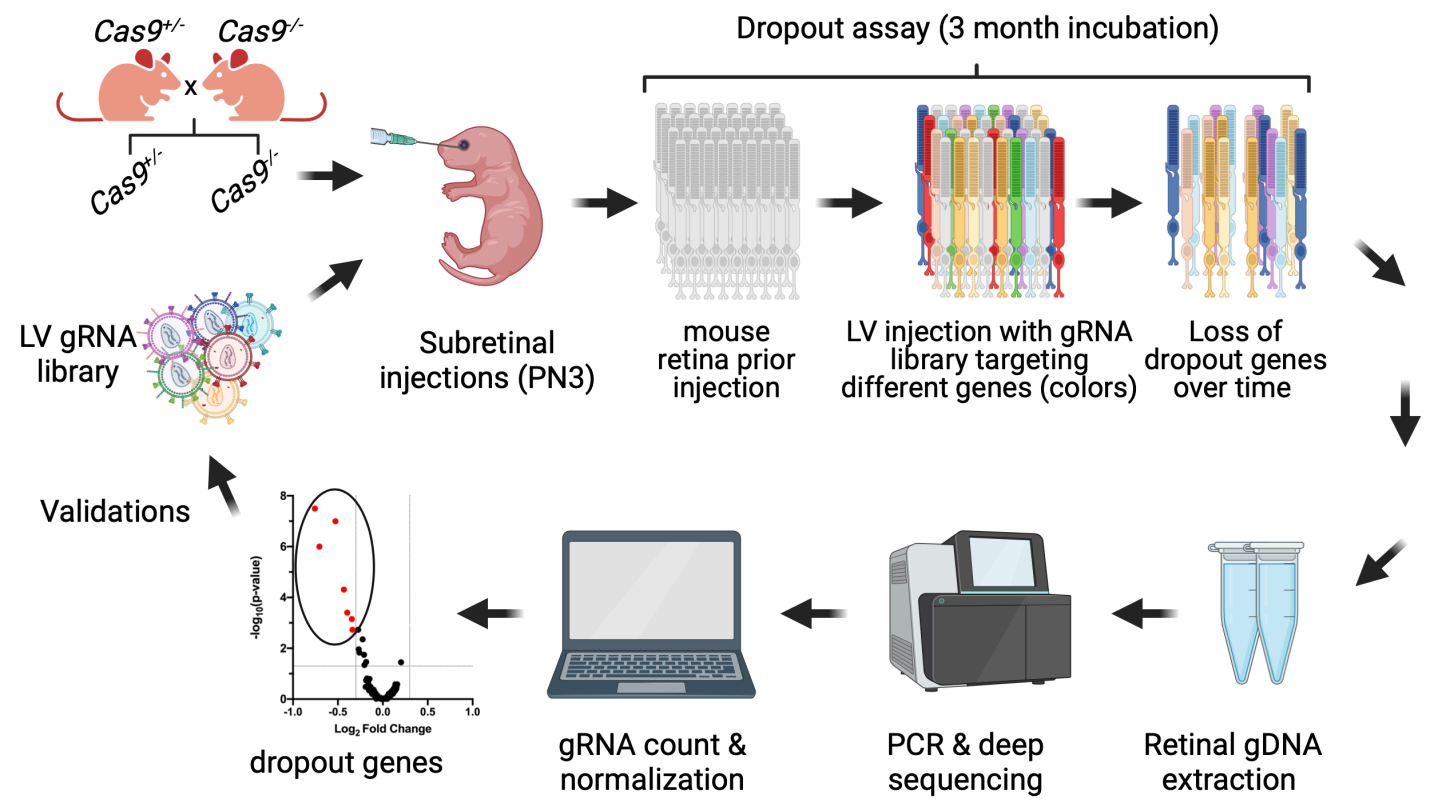
CRISPR knock-out screen experimental design. For this study, heterozygous transgenic mice carrying ubiquitously expressing CRISPR-Cas9 (Cas9^+/−^) and wild type mice were bred to ensure litters that included experimental (Cas9^+/−^) and control (Cas9^−/−^) mice. At PN3, mice were subretinally injected with a lentiviral (LV) CRISPR-KO library containing both targeting and non-targeting (control) gRNAs. The use of LV particles allowed for permanent integration and sustained expression of individual gRNAs within the genome of mouse photoreceptor cells (shown as photoreceptors of different colors), effectively serving as a genetic tag. The cell dropout was analyzed three months post injection, and the stable integration of gRNA sequences into the genome of photoreceptors enabled their detection by PCR amplification, followed by amplicon sequencing. In the data analysis steps (gRNA count and normalization), each gRNA was quantified relative to those in the control mice. If the expression of a particular gRNA was deleterious to the cells, those cells would die (dropout), depleting the gRNA sequence from the cell population.

After establishing the photoreceptor targeting efficiency and the optimal conditions for subretinal injections, we proceeded to the CRISPR knock-out screen experimental design, in which we aimed to use cell viability as the assay (i.e. dropout screen) (Fig. 2). First, to ensure sufficient and continuous expression of the Cas9 enzyme in the retinal cells we used a transgenic mice carrying ubiquitously expressing CRISPR-Cas9^43^ from the Gt(ROSA)26 locus^44^, further referred to as Cas9 mice. Breeding heterozygous Cas9^+/−^ mice with wild type (WT) mice ensured litters that included experimental (Cas9^+/−^) and control (Cas9^−/−^) mice. At PN3, mice were subretinally injected with a lentiviral (LV) CRISPR-KO library containing both targeting (experimental) and non-targeting (control) gRNAs, and the cell dropout was analyzed at PN90. The use of LV particles to deliver gRNA libraries allows for permanent integration and sustained expression of individual gRNAs within the host genome, effectively serving as a genetic tag. Under these settings, each transduced and edited photoreceptor can be considered as a “single-cell KO model”, whose survival will depend on how essential the target gene is, and on the effectiveness of the integrated gRNA. The stable integration of gRNA sequences into the gDNA of photoreceptors enabled their detection by PCR amplification of the gRNA cassette from the retinal gDNA and subsequent amplicon sequencing (Fig. 2). In the experimental condition (Cas9^+/−^ mice) we expected most of the targeting gRNAs to induce specific gene knockouts (targeting both alleles), therefore if expression of a particular gRNA was deleterious to the cells, those cells would die (dropout), depleting the gRNA sequence from the cell population. Thus, during the data analysis, we quantified each gRNA relative to those in the control mice (Fig. 2).

### Design and generation of the LV-KO gRNA library targeting ER and the UPS pathways

We generated a CRISPR-KO perturbation library containing ~500 gRNA sequences, targeting 63 retina-expressed genes (six gRNAs per gene), and 100 non-targeting controls (**Supplemental Table 1**). Fifty-five genes coded for members of the protein processing and stress response pathway of the endoplasmic reticulum (ER) or the ubiquitin-proteasome system (UPS). We chose to investigate this pathway as the impaired processing and clearing of misfolded proteins is one of the important causes of photoreceptor cell degeneration^45^. The remaining eight genes were known IRD disease genes serving as positive controls (*Aipl1*, *Cep290*, *Nmnat1*, *Pde6a*, *Pde6b*, *Prpf8*, *Rho*, *Ttc21b*).

The gRNAs were cloned as a pool into an expression vector containing the U6 promoter and gRNA scaffold (Fig. 1A). Through amplicon sequencing of the library, we confirmed the presence of all the designed gRNA sequences, 95% of which were within a 2-fold difference from the median sequence depth (**Supplemental Fig. 1**). We observed no significant differences between representation and distribution of targeting and non-targeting gRNAs.

### In vivo perturbation of the IRD, the ER, and the UPS pathway genes

**Figure 3:**
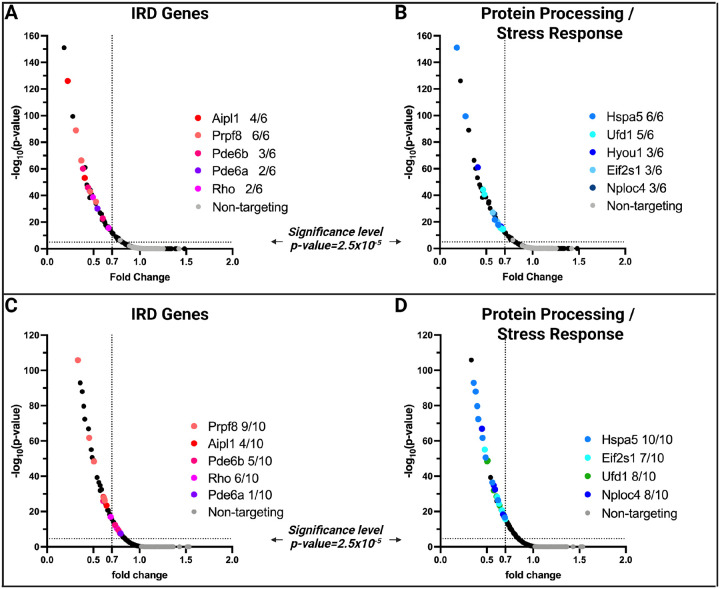
*In vivo* perturbation of the IRD, the ER and the UPS pathway genes. **A-B**. Sixty-three retina-expressed genes were perturbed in the primary screen, employing six targeting gRNAs/gene and 100 non-targeting control gRNAs. Considering a dropout rate of least 30% (i.e., Fold change 0.7) and more than one significant gRNA/gene, 10 genes were significantly depleted in the *Cas9*^+/−^ mice compared to the controls. None of the gRNAs showed enrichment in the Cas9^+/−^ mice, and only one non-targeting guide showed depletion of 20%. Of the 10 most significantly depleted genes, five were known non-syndromic IRD genes (*Aipl1*, *Pde6a*, *Pde6b*, *Prpf8*, *Rho*) **(A)**, while the other five were ER or UPS pathway genes (*Hspa5, Ufd1, Hyou1, Eif2s1, Nploc4*) **(B)**. **C-D**. Validation screen results. Validation was performed by perturbing 14 genes with 10 gRNAs/gene, corresponding to the most significant ER genes and all eight control IRD genes. One hundred non-targeting control gRNAs were also included in the library. The screen confirmed significant depletion of gRNAs targeting five non-syndromic IRD genes (*Aipl1*, *Pde6a*, *Pde6b*, *Prpf8*, *Rho*) **(C)**. Guide RNAs for six ER genes (*Hspa5, Nploc4, Eif2s1, Ufd1, Cul1*, and *Skp1*) showed significant depletion, with the strongest effect size and largest number of positive gRNAs detected for *Hspa5*, *Eif2s1, Nploc4*, and *Ufd1* (**D**).

Healthy *Cas9*^+/−^ (n = 7) and Cas9^−/−^ mice (n = 6) were used as experimental and control group, respectively. Five injected retinas were pooled per experimental unit (n = 1), and no inclusion/exclusion criteria were used. Fifty-five gRNAs showed a statistically significant dropout in the *Cas9*^+/−^ mice compared to the controls, which represented 21 genes (Table 1). However, when considering at least 30% dropout rate, only 35 gRNAs targeting 17 genes were significant, of which 10 genes had more than one significant gRNA/gene (Fig. 3, Table 1). This suggests that the gRNAs were efficient and most likely led to the knock-out of the gene that they were targeting, which in turn led to the photoreceptor cell death within 3 months after injection. None of the gRNAs showed enrichment in the Cas9^+/−^ mice, and only one non-targeting guide showed depletion of 20%.

Of the 10 most significantly depleted genes, five were known non-syndromic IRD genes (*Aipl1*, *Pde6a*, *Pde6b*, *Prpf8*, *Rho*)^14^, while the other five were ER or UPS pathway genes (Fig. 3A, Table 1). The most significantly depleted individual IRD gene gRNA targeted *Aipl1*, which is associated with a severe early-onset retinal disorder, Leber Congenital Amaurosis (LCA)^46^. However, on the gene level, *Prpf8* showed the strongest overall effect, with all six gRNAs targeting this gene significantly depleted (Table 1).

Only two gRNAs against each of the two syndromic ciliopathy genes: *Ttc21b*^47^ and *Cep290*^48^ showed significant depletion in the Cas9^+/−^ mice, and of these, only one gRNAs against *Ttc21b* exhibited more than 30% depletion (Table 1). None of the gRNAs targeting the positive control IRD gene *Nmnat1* showed a significant effect. Several ER and UPS pathway genes showed depletion, with *Hspa5*, encoding a heat-shock protein, being the most significant (Fig. 3B).

To validate our top hits, we performed a validation experiment in which we administered subretinally a smaller LV-KO gRNA library consisting of 140 gRNAs targeting the most significant ER genes, all eight control IRD genes (14 genes, 10 gRNAs/gene), and 100 non-targeting gRNAs (**Supplemental Table 2**).

Healthy *Cas9*^+/−^ (n = 10) and Cas9^−/−^ mice (n = 14) were used as experimental and control group, respectively. Five injected retinas were pooled per experimental unit (n = 1), and no inclusion/exclusion criteria were used. Validations confirmed significant depletion of gRNAs targeting five non-syndromic IRD genes (*Aipl1*, *Pde6a*, *Pde6b*, *Prpf8*, *Rho*), where again the dropout for the *Prpf8* and *Aipl1* gRNAs was most significant (Fig. 3C). For *Rho* and *Pde6b*, only one out of six (*Rho*) and five (*Pde6b*) significant gRNAs showed depletion of more than 30%, and only one gRNA for *Pde6a* showed depletion at all. Guide RNAs for six ER genes (*Hspa5, Nploc4, Eif2s1, Ufd1, Cul1*, and *Skp1*) showed significant depletion, with the strongest effect size and largest number of positive gRNAs detected for *Hspa5*, *Eif2s1, Nploc4*, and *Ufd1* (Fig. 3D, Table 1). None of the gRNAs targeting the positive control genes *Ttc21b, Cep290*, and *Nmnat1* showed significant dropout, although two guides for *Cep290* exhibited a depletion trend that did not reach statistical significance.

### Downregulation of specific retinal genes delays photoreceptor degeneration in a mouse model of IRD

Next, we wanted to understand if our approach can detect enrichment of gRNAs that may have a protective effect in a mouse model of IRD. To test this hypothesis, we chose a *Rho-Pro23His* knock-in model that shows a moderate rate of retinal degeneration^28^ and gRNAs against four retinal genes *Mir181a-1*, *Mir181b-1*, *Sarm1*, *Tsc2*, whose downregulation was demonstrated to delay photoreceptor loss in IRD mouse models^49–51^. These gRNAs were incorporated into the gRNA library targeting ER genes.

To achieve successful genome editing, heterozygous *Rho-Pro23His* mice were crossed with homozygous Cas9 mice to generate experimental *Rho-Pro23His-Cas9*^+/−^ and control *Cas9*^+/−^ mice in one litter. We compared two Cas9-expressing genotypes to identify gRNAs that either alleviate or exacerbate photoreceptor degeneration in the *Rho-Pro23His* background relative to the WT *Rho* genotype. *Rho-Pro23His-Cas9*^+/−^ (n = 4) and Cas9^+/−^ mice (n = 7) were used as experimental and control group, respectively. Five injected retinas were pooled per experimental unit (n = 1), and no inclusion/exclusion criteria were used. The LV libraries were administered subretinally at PN3, and the retinas were harvested and processed as before at PN90.

**Figure 4:**
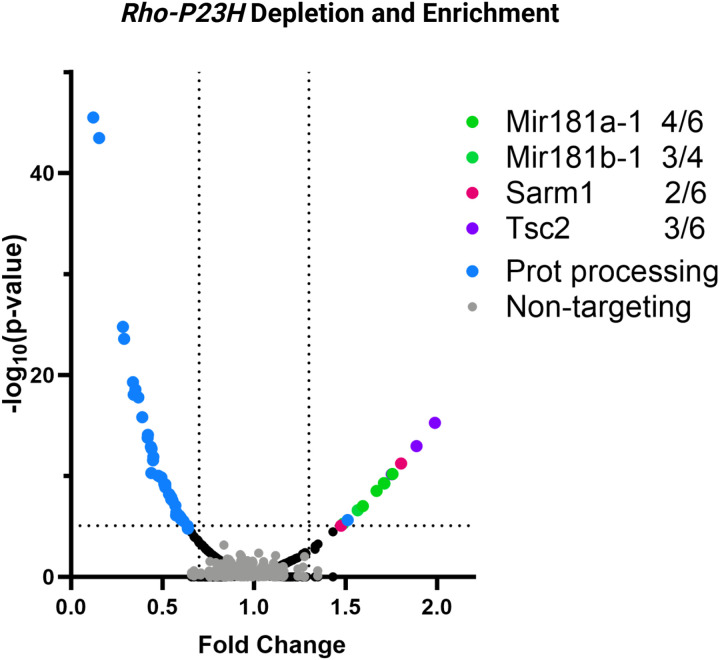
Downregulation of specific retinal genes delays photoreceptor degeneration in a mouse model of IRD. Heterozygous *Rho-Pro23His* mice were crossed with homozygous Cas9 mice to generate experimental *Rho-Pro23His-Cas9*^+/−^ and control *Cas9*^+/−^ mice. Mice were subretinally injected at postnatal day 3 using the gRNA library targeting ER genes, supplemented with gRNAs targeting four retinal genes (*Mir181a-1*, *Mir181b-1*, *Sarm1*, *Tsc2*) whose downregulation was demonstrated to delay photoreceptor loss in IRD mouse models. Twelve gRNAs targeting these four genes were found enriched in the *Rho-Pro23His-Cas9*^+/−^ retinas compared to control *Cas9*^+/−^ mice, corroborating previous findings. In addition, multiple gRNAs against ER protein processing genes (highlighted as blue dots) showed a deleterious effect in the *Rho-Pro23His* mice as well.

We observed that 12 gRNAs targeting *Mir181a-1*, *Mir181b-1*, *Sarm1*, or *Tsc2* were enriched in the *Rho-Pro23His-Cas9*^+/−^ retinas compared to control *Cas9*^+/−^ mice, corroborating previous findings^49–51^. Additionally, multiple gRNAs against seven ER genes (*Eif2s1, Hspa5, Hspa8, Nploc4, Hyou1, Ufd1*, and *Vcp*) showed a deleterious effect in the *Rho-Pro23His* mice. Guide RNAs targeting four of these genes (*Eif2s1, Hspa5, Nploc4, Ufd1*) also led to cell depletion in the WT mice, but this effect was further enhanced on the *Rho-Pro23His* genetic background (Fig. 4).

## DISCUSSION

In this study, we describe the development of an *in-vivo* CRISPR-based KO perturbation screen methodology to investigate essential genes in the retina and genetic modifiers of retinal degeneration. As proof of concept, we used gRNAs targeting eight known retinal degeneration genes, and additionally, we tested the importance of 55 genes involved in the ER or UPS pathways. Primary screen with six gRNAs per gene, followed by a validation screen with ten gRNAs per gene, both with a 3-month interval post-injection, showed significant depletion of gRNAs targeting five IRD genes (*Aipl1*, *Pde6a*, *Pde6b*, *Prpf8*, *Rho*) and six ER/UPS genes (*Hspa5, Nploc4, Eif2s1, Ufd1, Cul1*, and *Skp1*). We did not observe significant depletion or enrichment for any of the 100 non-targeting control gRNAs (negative controls). Altogether, these findings demonstrate the feasibility of performing *in-vivo* CRISPR screening in the mouse retina.

Of the five IRD genes with significant effects, three (*Aipl1*, *Pde6a*, *Pde6b*) are associated with a recessive retinal degeneration in humans^52–54^. *Aipl1* encodes Aryl Hydrocarbon Receptor-Interacting Protein-like 1, which is essential for photoreceptor development and function^55^. *Pde6a* and *Pde6b* encode subunits of rod photoreceptor phosphodiesterase 6 (Pde6), a key enzyme in the rod photoreceptor phototransduction pathway^56,57^. Bi-allelic loss-of-function variants in *AIPL1, PDE6A*, and *PDE6B* cause early-onset recessive retinal degeneration^55–57^; therefore, significant depletion of gRNAs targeting these genes was anticipated. We also observed a significant depletion of gRNAs targeting the *Rhodopsin* (*Rho*) gene. Despite being mostly observed in dominant retinitis pigmentosa, it has also been reported in recessive cases, and photoreceptor cell loss due to *Rho* disruption is corroborated by the *Rho* knockout models^58,59^. Among all the tested IRD genes, the strongest effect was observed for *Prpf8*, with 6 out of 6 significant gRNAs in the primary screen and 9 out of 10 significant gRNAs in the validation screen. Among the five significant genes, *PRPF8*, which encodes a pre-mRNA splicing factor, was the only one associated exclusively with a dominant phenotype in humans^60^. However, this is most likely due to embryonic lethality, as *PRPF8* is one of the most constrained genes in the human genome^4,61^ and was established as a core fitness gene in cell culture screens^62^.

For three tested IRD genes, we observed a modest effect limited to a few gRNAs that did not validate in the secondary screen (*Ttc21b* and *Cep290*) or no significant dropout for any of the tested gRNAs (*Nmnat1*). Proteins encoded by *Ttc21b* and *Cep290* are crucial for the function and maintenance of cilia^47,63^. Consequently, pathogenic variants in these genes can result in multisystemic diseases known as ciliopathies or cause isolated retinal phenotypes in human patients^47,63^. One of the distinct features of the *CEP290*-associated retinal degeneration is a relatively good preservation of the photoreceptor nuclear layer, even though the photoreceptors are nonfunctional^64^. This may explain why *Cep290* disruption did not result in a significant photoreceptor dropout three months after the gRNA administration; possibly a longer incubation may be necessary to observe a significant effect. We expect the same to be true for *Ttc21b*.

Surprisingly, none of the gRNAs targeting *Nmnat1* showed any dropout in our screen. *Nmnat1* encodes Nicotinamide Mononucleotide Adenylyltransferase 1, which is an essential enzyme in NAD+ (nicotinamide adenine dinucleotide) biosynthesis in the nucleus of all cells^65^. Pathogenic variants in *NMNAT1* have been associated with early-onset retinal degeneration^66,67^, and mice homozygous for one of the variants identified in patients (p.V9M) also show significant defects by 1 month of age^68^. However, *NMNAT1* has not been identified as one of the core fitness genes in other screens, nor is it a constrained gene in the human genome^4,61,62^. Therefore, some redundancy for the NMNAT1 function may be possible, or a longer incubation period after the gRNA library injections is necessary to observe the effect. A significant reason for the discrepancy between patient data and mouse models may be that, in both patients and mice, the genetic defect is present from the start and influences retinal development before birth. In contrast, in our system, by the time the lentiviral vector (LV) expresses the gRNAs and these act on the targeted gene, the cells are post-mitotic, and the essential gene product is already present in the cell. Only after this gene product is depleted can we start to observe the effect.

All of the significant ER/UPS genes (*Hspa5, Nploc4, Eif2s1, Ufd1, Cul1*, and *Skp1*) were also shown to be core essential genes in human cell lines^62^. Of all of the ER/UPS genes we targeted in our screen, three additional genes were previously established as core fitness genes: *Sec13*, *Vcp*, and *Hyou1*^62^. In our primary screen, we observed a significant depletion for only 1–2 gRNAs for these genes, but they were not included in the validation screen.

We also investigated the utility of the CRISPR perturbation screen to find possible genetic modifiers of retinal phenotypes. We chose ER/UPS protein processing genes as targets because defects in protein chaperones and other ER components involved in the stabilization of misfolded proteins result in proteasome overload and subsequent cellular stress, which is one common cause of photoreceptor degeneration in IRDs^45,69^. To test the hypothesis that depletion of the ER or UPS genes may affect IRD progression, we chose a mouse model of a relatively common Rhodopsin Pro23His mutation, which was previously shown to cause protein misfolding, accumulation in the ER stress, and ultimately photoreceptor cell death^70–73^. Depletion of seven genes (*Eif2s1, Hspa5, Hspa8, Nploc4, Hyou1, Ufd1*, and *Vcp*) showed an exacerbating effect on the *Rho-Pro23His* retinal degeneration compared to the *Cas9*^+/−^ mice. Notably, all of these genes except *Hspa8* have been described as core fitness genes in other screens^62^. Given that these genes are involved in protein folding and ER stress response (*Eif2s1, Hspa5, Hspa8, Hyou1*, and *Ufd1*) and protein degradation/ubiquitin pathway (*Nploc4* and *Vcp*)^74^, their influence on IRD progression is not surprising. Our library also included positive control gRNAs against genes (*Mir181a-1*, *Mir181b-1*, *Sarm1*, *Tsc2*), whose downregulation has been demonstrated to delay photoreceptor loss in IRD mouse models^49–51^. gRNAs against the positive controls showed enrichment, which further validates our methodology as a tool for finding positive and negative modifiers of retinal cell death. Although in this case we did not perform a separate validation experiment, the robustness of the data, supported by the number of biological replicates in each animal group (4 P23H/Cas9 vs 7 Cas9+/−, corresponding to 16 vs 28 mice, respectively), the number of targeting guides per gene (average of 3 gRNAs per gene), and prior functional evidence about these genes^49–51^, support the validity of the findings.

CRISPR screens have been widely explored on *in-vitro* models but significantly less *in-vivo*^75^. This study demonstrates the feasibility of performing a CRISPR screen in a mouse retina, which is crucial for identifying new retinal-specific genes and modifiers of retinal disease. However, there are several limitations of this study. First, the number of photoreceptors that can be transduced with a single LV injection, combined with the very narrow window during which these neuronal cells are receptive to lentiviral infection (up to post-natal day 3), does not allow for the simultaneous and reliable perturbation of all protein-coding genes in a single experiment. Although a genome-wide perturbation in the retina has been recently attempted in a similar study^76^, we estimate that up to 150 genes can be reliably screened in one library. If a larger or a smaller library is desired, more or fewer retinas may be pooled; however, in our experience, to diminish the experimental variability related to subretinal injections, we advise combining at least four retinas into one biological sample. Also, by the time the gRNAs are expressed from the LVs and the target genes are disrupted, the cells are post-mitotic and therefore only pathways relevant to the post-mitotic cells can be studied with the current assay. Additionally, it is important to highlight that the use of murine animal models for *in-vivo* screens may restrict the discovery of novel genotype-phenotype associations to rod-predominant retinal disorders, as over 90% of the mouse neural retina is made of this photoreceptor cell type.

Despite these limitations, we showed that our *in-vivo* perturbation assay in the retina is an effective approach for thoroughly investigating retinal pathways and their association with photoreceptor survival. This is particularly timely given the large number of genes still lacking functional characterization in both physiological and disease contexts. Therefore, we believe that *in-vivo* CRISPR-based perturbation studies have great potential to lead to the discovery of novel therapeutic targets, which may be either gene-agnostic or specific to the primary genetic defects.

## MATERIALS AND METHODS

### Ethical statement

This study conformed to the Association for Research in Vision and Ophthalmology Statement for the Use of Animals in Ophthalmic and Vision Research, and all procedures were approved by the Animal Care and Use Committee of the Schepens Eye Research Institute. The study was also reported in accordance with the ARRIVE guidelines (https://arriveguidelines.org).

### Animal Husbandry

Wild-type albino CD1 mice were purchased from Charles River Laboratories (Wilmington, MA), while knock-in mice carrying ubiquitously expressing CRISPR-Cas9 from the Gt(ROSA)26 locus (Cas9+/−)^43^ were purchased from Jackson Laboratory (Bar Harbor, ME). The *Rho-Pro23His* knock-in model was instead received from a collaborator (courtesy of Prof. Qin Liu, MEE, Boston, MA.)

Mice were bred and maintained in the Schepens Eye Research Institute Animal Care Facility where they were fed ad libitum irradiated pelleted diet (LabDiet, MO) and water ad libitum and housed in a 14-hour light/10-hour dark cycle.

### Genotyping

Genotyping for the presence of the Cas9 allele was determined by gel electrophoresis after DNA was amplified using a multiplex PCR strategy. A common forward primer, 5’-CAGTAAGGGAGCTGCAGTGG-3’, located in the first intron of the Gt(ROSA)26 locus, was used to amplify both the wildtype and knock-in alleles. The reverse primer for the wildtype allele, 5’-CCGAAAATCTGTGGGAAGTC-3’, was also located in intron 1, and this primer pair specified a 301 bp amplicon in the wildtype allele. For the knock-in allele, the reverse primer, 5’- TGCCAAGTGGGCAGTTTACC-3’, was located within the inserted fragment and specified a 435 bp amplicon. Genotyping of the Rho-P23H allele was also determined by PCR, using two primers, 5’- TGGAAGGTCAATGAGGCTCT −3’ and 5’- GACCCCACAGAGACAAGCTC −3’, which specified a 399 bp amplicon in the mouse wild-type allele and a 573 bp amplicon in the transgenic P23H allele^77^.

The 10 μl PCR reactions had final concentrations of 100 μmol/L for each primer, 200 nmol/L for each of the dNTPs, 2 mmol/L MgCl2, and 1 unit of Hot FirePol DNA polymerase (Solis BioDyne, Tartu, Estonia). The thermocycling protocol was 95 °C for 14 min; 30 cycles of 95 °C for 45 s, 63 °C for 30 s and 72 °C for 30 s; 72 °C for 10 min.

### CRISPR-KO gRNA library generation

Fifty-five genes that were members of the ER protein processing and stress response pathway and the UPS pathway were chosen based on the public databases (KEGG pathway mmu04141). Eight known retinal disease genes (*Aipl1*, *Cep290*, *Nmnat1*, *Pde6a*, *Pde6b*, *Prpf8*, *Rho, Ttc21b*) were chosen as positive controls. Four genes (*Mir181a-1*, *Mir181b-1*, *Sarm1*, *Tsc2*) were chosen for their protective effect on IRD when downregulated. For each gene, six gRNAs of 21bp were designed using the CRISPick tool from Broad Institute (portals.broadinstitute.org/gppx/crispick/public)^78,79^. The only exception was *Mir181b-1*, for which CRISPick designing tool only returned four gRNAs. This strategy generated a library of 400 targeting gRNAs, and 100 non-targeting gRNAs were finally included as negative control. The complete list of all gRNAs can be found in **Supplemental Table 1**.

Flanking 60-bp long oligonucleotide arms at 5’ (GTAACTTGAAAGTATTTCGATTTCTTGGCTTTATATATCTTGTGGAAAGGACGAAACACC) and 3’ (GTTTTAGAGCTAGAAATAGCAAGTTAAAATAAGGCTAGTCCGTTATCAACTTGAAAAAGT) were added to each gRNA sequence, thus generating a 141-long oligo pool, which was purchased on Twist Bioscience.

We obtained the lentiviral CRISPR-KO vector pXPR_BRD043 by the Genetic Perturbation Platform (GPP) at the Broad Institute. We modified that vector by replacing the EIF1a promoter upstream the mCherry reporter with a Rhodopsin Kinase promoter, and used this modified version to clone the oligo pool by Gibson Assembly with a strategy adapted from a previously published protocol^80^. A similar strategy was adopted to generate our validation library, except that in this case ten gRNAs were designed per each candidate gene (**Supplemental Table 2**).

### Lentivirus generation

The day before the transfection, 7×10^6^ HEK-293T cells were seeded in a T-150 flask. The day after, twelve micrograms of the cloned gRNA library were co-transfected in a ratio of 3:2:1 with two second generation lentiviral plasmids expressing packaging (psPAX2, Addgene plasmid #12260) and envelope (pMD2.G, Addgene plasmid #12259) components using the Fugene HD protocol (Promega, Cat. # E2311). Forty-eight hours post transfection, the media was collected and ultracentrifuged at 4°C for two hours at 25,000 rpm using the Optima X-100 Beckman Coulter, rotor SW41).

After ultracentrifugation, the supernatant was aspirated, and the pellet was resuspended in 100ul sterile NaCl 0.9% sterile. Tubes were sealed with parafilm and left resuspending at 4°C O/N. The day after, 10ul aliquots were made, which were stored at −80°C.

Lentivirus was titrated using the Lenti-X qRT-PCR Titration Kit (Takara) according to the manufacturer’s instructions.

### Subretinal Injections

Mice were subretinally injected at PN3 with lentiviral particles (LVs) containing either fluorescent reporters or the gRNA library and using a 33G blunt end needle (Hamilton, Reno, NV). Each mouse received only one injection, which was performed in a dedicated Biosafety Level 2 (BSL-2) room located in the animal facility and with the use of a stereoscopic microscope. Mice were anesthetized by hypothermia, by being placed in a latex glove on ice for up to 4 minutes. Post-injection recovery was achieved by using a heating pad (32–35°C) to gradually warm the mice before being reintroduced in a disposable hazardous cage with their mother. Three days after the injections, mice and their mothers were transferred to a regular, non-disposable, cage.

Retinas injected with fluorescent markers were harvested at PN21 and PN90 and analyzed by flow cytometry to assess transduction efficiency. Retinas injected with the gRNA library were harvested at PN90 for subsequent genomic DNA extraction.

### Genomic DNA extraction and downstream molecular analysis

Mice were euthanized using a carbon chamber according to the NIH ARAC guidelines. Injected eyes were enucleated, cornea was pierced, and the whole eyes were incubated in HBSS 1X without Calcium and Magnesium (Gibco Cat.14175–095) for 30 minutes to ensure sufficient detachment of the retina from the RPE. Subsequently the retinas were dissected under a stereomicroscope, incubated in the STE buffer (Tris, EDTA 0.5M, 1% SDS, NaCl, pH 8.5) and the gDNA was extracted using an already published protocol^81^.

### Next-generation sequencing data analysis and statistics

Retinal genomic amplification was performed using primers flanking the integrated gRNA and Titanium^®^ Taq DNA Polymerase (Takara, Cat. # 639242). For each sample, 20μg were amplified at low (25) cycle number, split in 8 PCR reactions of 2.5μg input each, in order to increase the diversity. The obtained amplicons were then purified using a PCR clean and concentrator (Zymo Research), quantified using Qubit 1x dsDNA high sensitivity kit (Q33231; Thermofisher, Waltham, MA), and 750–1000ng total DNA was used as input for NGS library preparation. NEBNext Ultra II DNA Library Prep kit (E7103; New England Biolabs, Ipswich, MA) was used to ligate Illumina adapters onto DNA followed by size selection for final library size of ~280bp. Libraries were multiplexed by adding 8bp indexes (E6440; New England Biolabs, Ipswich, MA) during the amplification step. Library quality was assessed by Tape Station 4200 HS D100 (5067–5584; Agilent, Santa Clara, CA) and quantified using Qubit HS D1000 before normalizing all libraries to 4nM concentration with a final loading concentration of 10pM after pooling. Sequencing was performed on an Illumina MiSeq instrument with v3 flow cell and a 150-cycle reagent kit (MS-102–3001; Illumina, San Diego, CA) with 1×150 single reads to achieve the desired coverage.

The gRNA enrichment or depletion was conducted using the PinAPL-Py software^82^.

## Supplementary Material

Supplementary Files

This is a list of supplementary files associated with this preprint. Click to download.

- SupplemetaryTables.xlsx
- SupplementalFig.1.png

## Figures and Tables

**Table 1.** List of significant gRNAs detected in either primary and validation screen.

Genes	Primary Screen (6 gRNAs/gene)			Validation screen (10 gRNAs/gene)		
	No. significant gRNAs		Total significant gRNAs	No. significant		Total significant gRNAs
	Dropout ^3^30%	Dropout <30%		Dropout ^3^30%	Dropout <30%	
***Aipl1***	2	2	4	3	1	4
*Atf4*		1	1	*Not included*		
*Cep290*		2	2			
*Cul1*	1		1	1	3	4
*Dnajc5*	1	1	2	*Not included*		
***Eif2s1***	2	1	3	5	2	7
***Hspa5***	4	2	6	9	1	10
*Hspa8*		2	2	*Not included*		
*Hyou1*	2	1	3	*Not included*		
*Lman1*		1	1	*Not included*		
***Nploc4***	2	1	3	5	3	8
***Pde6a***	2		2		1	1
***Pde6b***	3		3	1	4	5
***Prpf8***	4	2	6	5	4	9
*Rbx1*	1	2	3	*Not included*		
***Rho***	2		2	1	5	6
*Sec13*	1		1	*Not included*		
*Skp1*	1		1	1	3	4
*Ttc21b*	1	1	2			
***Ufd1***	5		5	6	2	8
*Vcp*	1	1	2	*Not included*		

## Data Availability

The datasets generated and analyzed during the current study are available in the Sequence Read Archive (SRA) repository, BioProject ID PRJNA1336802, BioSamples SAMN52091418-SAMN52091458, submitted on 09-30-2025 by R. Sangermano (Riccardo_Sangermano@MEEI.HARVARD.EDU).
